# A motion-corrected deep-learning reconstruction framework for accelerating whole-heart magnetic resonance imaging in patients with congenital heart disease

**DOI:** 10.1016/j.jocmr.2024.101039

**Published:** 2024-03-22

**Authors:** Andrew Phair, Anastasia Fotaki, Lina Felsner, Thomas J. Fletcher, Haikun Qi, René M. Botnar, Claudia Prieto

**Affiliations:** a School of Biomedical Engineering and Imaging Sciences, King’s College London, London, United Kingdom; b School of Biomedical Engineering, Shanghai Tech University, Shanghai, China; c Instituto de Ingeniería Biológica y Médica, Pontificia Universidad Católica de Chile, Santiago, Chile; d Escuela de Ingeniería, Pontificia Universidad Católica de Chile, Santiago, Chile; e Millennium Institute for Intelligent Healthcare Engineering, Santiago, Chile; f Technical University of Munich, Institute of Advanced Study, Munich, Germany

**Keywords:** Congenital heart disease, Cardiac MRI, Image reconstruction, Convolutional neural network, 3D whole-heart, Motion correction

## Abstract

**Background:**

Cardiovascular magnetic resonance (CMR) is an important imaging modality for the assessment and management of adult patients with congenital heart disease (CHD). However, conventional techniques for three-dimensional (3D) whole-heart acquisition involve long and unpredictable scan times and methods that accelerate scans via k-space undersampling often rely on long iterative reconstructions. Deep-learning-based reconstruction methods have recently attracted much interest due to their capacity to provide fast reconstructions while often outperforming existing state-of-the-art methods. In this study, we sought to adapt and validate a non-rigid motion-corrected model-based deep learning (MoCo-MoDL) reconstruction framework for 3D whole-heart MRI in a CHD patient cohort.

**Methods:**

The previously proposed deep-learning reconstruction framework MoCo-MoDL, which incorporates a non-rigid motion-estimation network and a denoising regularization network within an unrolled iterative reconstruction, was trained in an end-to-end manner using 39 CHD patient datasets. Once trained, the framework was evaluated in eight CHD patient datasets acquired with seven-fold prospective undersampling. Reconstruction quality was compared with the state-of-the-art non-rigid motion-corrected patch-based low-rank reconstruction method (NR-PROST) and against reference images (acquired with three-or-four-fold undersampling and reconstructed with NR-PROST).

**Results:**

Seven-fold undersampled scan times were 2.1 ± 0.3 minutes and reconstruction times were ∼30 seconds, approximately 240 times faster than an NR-PROST reconstruction. Image quality comparable to the reference images was achieved using the proposed MoCo-MoDL framework, with no statistically significant differences found in any of the assessed quantitative or qualitative image quality measures. Additionally, expert image quality scores indicated the MoCo-MoDL reconstructions were consistently of a higher quality than the NR-PROST reconstructions of the same data, with the differences in 12 of the 22 scores measured for individual vascular structures found to be statistically significant.

**Conclusion:**

The MoCo-MoDL framework was applied to an adult CHD patient cohort, achieving good quality 3D whole-heart images from ∼2-minute scans with reconstruction times of ∼30 seconds.

## Graphical abstract

**Figure:**
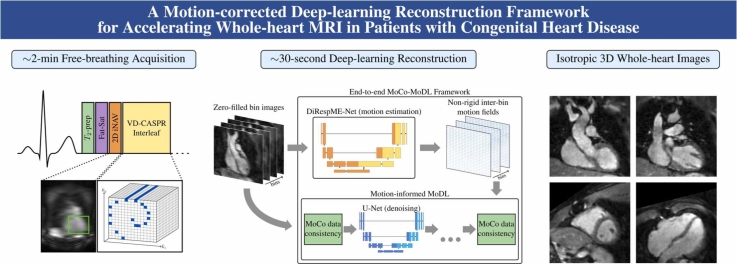

## 1 Background

Cardiovascular magnetic resonance (CMR) is well-established for anatomical assessment, procedure planning, and the management of patients with congenital heart disease (CHD) [1], [2], [3]. It is non-invasive, free of ionizing radiation, and considered to be the gold-standard imaging modality for the assessment of ventricular volume and myocardial mass [4], [3]. However, conventional whole-heart CMR acquisition strategies rely on diaphragmatic respiratory gating [5] and electrocardiogram (ECG) triggering, leading to long, unpredictable scan times with poor scan efficiency since data acquired outside a small respiratory window are rejected.

Two-dimensional (2D) and three-dimensional (3D) image-based navigators (iNAVs) have been proposed as an alternative to diaphragmatic navigators [6], [7], [8], [9], [10], [11], [12], [13] and have been successfully applied to adult CHD patients [10]. By including iNAVs in imaging sequences, motion registration algorithms can be applied to low-resolution navigator images to obtain 2D or 3D motion curves. These can then be utilized to apply translational motion correction [7], [8], [9], [10] or allow respiratory binning for non-rigid (NR) motion estimation and correction [11], [12], [13], [14], [15], enabling 100% respiratory scan efficiency and thus shorter scans.

Even shorter scan times have been achieved by utilizing undersampled Cartesian or non-Cartesian k-space trajectories. To avoid undersampling artifacts, these approaches often combine parallel imaging and variable-density or non-Cartesian sampling with the use of iterative reconstruction methods incorporating compressed sensing [16] or low-rank regularization terms [17], [18], [15]. Bustin et al. [15] proposed a technique that combined 2D iNAVs for respiratory binning, an undersampled variable-density spiral-like Cartesian trajectory [19], [18], NR inter-respiratory-bin motion correction [14] and patch-based low-rank reconstruction (PROST) [18] to achieve whole-heart coronary magnetic resonance angiography (CMRA) scans with 0.9 mm isotropic resolution in 5–10 minutes. This approach, called NR-PROST, was originally applied to healthy subjects and patients with suspected coronary artery disease and has since also been validated in a CHD patient cohort [20]. However, the NR motion estimation and subsequent iterative reconstruction steps come at the cost of clinically infeasible reconstruction times; Bustin et al. [15] reported that the reconstruction of each 3D whole-heart image took ∼50 minutes.

More recently, deep neural networks have been proposed for CMR image reconstruction [21], [22], [23], [24], [25], [26]. While compressed sensing and low-rank techniques impose a priori information during the reconstruction, supervised deep-learning-based reconstruction methods instead learn regularizing information from a large set of fully sampled training data. Thus, the sensitivity of the reconstruction to the hand-chosen transform domain(s) and weighting parameter(s) is removed. Another advantage of deep-learning networks is that the computational burden is shifted to the training of neural network parameters rather than the reconstruction itself; once the network is trained, the reconstruction during inference is generally fast [21].

Various network architectures and frameworks have been proposed for 3D CMR reconstruction. These include techniques that approach the problem as one of k-space interpolation [27], super-resolution techniques that obtain high-resolution images from lower-resolution scans [23], [24], and techniques that operate in image space to remove noise and artifacts from the image [25], [26], [28], [29], [30], while (optionally) also enforcing data consistency (DC) [26], [28], [29], [30]. For instance, Fuin et al. [26] proposed an approach that combined the acquisition sequence and iNAV-based translational respiratory motion correction of Bustin et al.’s [15] framework with a multi-scale variational neural network that took the place of the 3D-PROST denoising. This was subsequently extended successfully for bright- and black-blood imaging of CHD patients [31]. However, in this technique, NR respiratory motion was not corrected for.

To incorporate NR motion within a deep neural network reconstruction, Qi et al. [29] proposed a motion-corrected model-based deep learning (MoCo-MoDL) reconstruction framework, which combined two deep-learning networks trained in an end-to-end fashion for 3D CMRA data. The first network estimated diffeomorphic NR motion fields from zero-filled input images [32], [33], [34], while the second utilized these fields in a motion-corrected model-based [35] reconstruction. The training set consisted of fully sampled healthy-subject data and two-to-three-fold undersampled patient data. A motion-corrected reconstruction was applied to this data to generate “ground truth” images, from which the corresponding undersampled k-space was synthetically simulated. Once trained, the MoCo-MoDL framework was applied to seven-fold-undersampled ∼2.5-minute acquisitions. Qi et al. [29] reported reconstruction times of ∼22 seconds for 3D images with 1.2 mm isotropic spatial resolution.

In this work, we propose to adapt, train, and validate the MoCo-MoDL framework for a cohort of adult CHD patients, to obtain whole-heart 3D images with 1.5 mm isotropic spatial resolution from a ∼2-minute scan with clinically feasible reconstruction times. Instead of generating training data via model-based k-space simulations from reconstructed images, a scheme for retrospectively undersampling acquired k-space data while preserving realistic sampling density profiles is implemented. This avoids the mismatch between training and test data that can otherwise arise from simulated k-space data. Corresponding reference images are generated using NR-PROST. The network is trained in an end-to-end manner using 39 CHD patient datasets acquired with three-or-four-fold undersampling (retrospectively undersampled to seven-fold) and tested on 8 CHD patient datasets acquired with prospective seven-fold undersampling.

## 2 Methods

### 2.1 Acquisition sequence

**Fig. 1:**
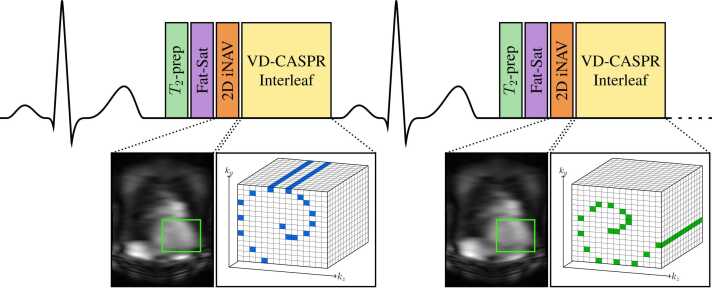
The ECG-triggered VD-CASPR bSSFP sequence. Following the trigger delay, one spiral-like interleaf of k-space frequency encodes is acquired at each heartbeat. Each acquisition is preceded by a *T*_2_-preparation pulse, a fat-saturation pulse, and a 2D iNAV acquisition. 2D, two-dimensional; bSSFP, balanced steady-state free precession; ECG, electrocardiogram; iNAV, image-based navigators; VD-CASPR, variable density Cartesian acquisition with spiral profile order.

A free-breathing ECG-triggered balanced steady-state free precession (bSSFP) sequence was utilized, as has been previously described for CMRA applications [18]. The sequence is depicted in Fig. 1. At each heartbeat, the k-space acquisition was preceded by a *T*_2_-preparation pulse, a fat-saturation pulse, and the acquisition of a low-resolution 2D iNAV [7]. A variable density Cartesian acquisition with spiral profile order (VD-CASPR) [19], [18] interleaf, which consisted of a series of frequency-encoded *k*_*x*_ readouts forming a spiral-like pattern in the *k*_*y*_-*k*_*z*_ plane, was acquired during each acquisition window. The number and location of frequency encodes acquired at each heartbeat were dependent on the subject’s RR interval and the desired overall undersampling factor, and were selected automatically to ensure a fully sampled k-space center [19], [18].

### 2.2 MoCo-MoDL reconstruction framework

**Fig. 2:**
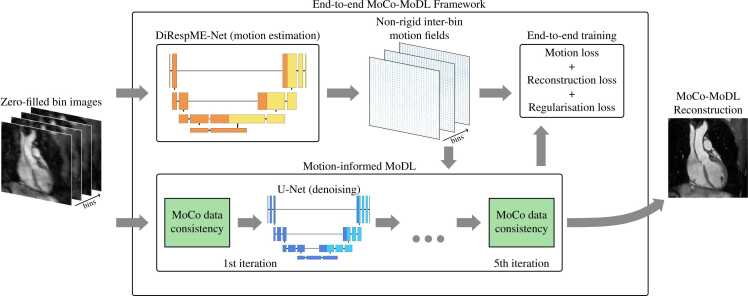
Schematic of the MoCo-MoDL reconstruction framework. Zero-filled respiratory-bin images are passed to the DiRespME-Net, which outputs non-rigid inter-bin motion fields. These fields are utilized in a motion-corrected reconstruction which alternates between a data consistency step and a denoising U-Net. During end-to-end training, the loss term includes both the motion loss, measured between the end-expiration-bin reference image and the remaining bin images warped to the end-expiration respiratory phase by the DiRespME-Net motion fields, and a reconstruction loss, measured between the reconstructed and reference images. DiRespME-Net, diffeomorphic respiratory motion estimation network; MoCo-MoDL, motion-corrected model-based deep-learning.

The MoCo-MoDL [29] reconstruction framework, as depicted in Fig. 2, was used to reconstruct 3D whole-heart images given the input of four zero-filled respiratory-bin images. As described below, the framework incorporated two convolutional neural networks. The first, a diffeomorphic respiratory motion estimation network (DiRespME-Net) [34], was used to obtain NR motion field estimates, while the second applied image denoising within an iterative motion-corrected reconstruction.

In practice, the 3D zero-filled images were split into overlapping 3D patches in the fully sampled readout direction before input into the MoCo-MoDL framework, due to memory constraints of the end-to-end training. Since undersampling artifacts do not propagate in fully sampled directions, this choice of patch direction prevents artifacts extending between adjacent patches, as they would were patching implemented in the undersampled phase-encoding directions. At the end of the reconstruction process, the 3D patches were recombined to provide a full-FOV (field of view) 3D image. This was achieved by discarding an edge layer of voxels in each 3D patch and averaging the remaining overlapping voxels.

#### 2.2.1 Zero-filled respiratory-bin reconstruction

Initially, the 2D iNAVs were registered to obtain a foot-head (*x*) and left-right (*y*) motion position for every heartbeat [7]. Each k-space readout was corrected for translational respiratory motion by applying the phase shift

(1) $${\overset{ˆ}{b}}_{jm}=b_{jm}\exp\left(2\pi ik_{jm}\cdot T_{m}\right),$$

where *b*_*jm*_ is the acquired k-space sample in position *j* along the *m*th readout, ${\overset{ˆ}{b}}_{jm}$ is the corrected value, **k**_*jm*_ is the 2D position vector representing the *k*_*x*_ and *k*_*y*_ coordinates of that sample, and **T**_*m*_ is the 2D translation vector representing the difference between the mean motion position over all heartbeats and the specific motion position obtained for the *m*th readout. This has the effect of aligning the position of the heart between respiratory bins, as well as increasing the image sharpness of each bin image.

The foot-head motion signal was used to bin the heartbeats into four equally populated respiratory bins with soft-gating [36], [14], this number having previously been demonstrated to be sufficient for NR respiratory-motion correction in whole-heart imaging [14]. A zero-filled reconstruction was performed for each bin, implemented as

(2) $$\rho_{ZF}=S^{*}F^{-1}U^{T}b$$

where, for *N* voxels, *N*_*b*_ respiratory bins, *N*_*c*_ coils, and *K*_*i*_ k-space samples in the *i*th respiratory bin, $b\in C^{N_{c}\left(\sum_{i=1}^{N_{b}}K_{i}\right)\times 1}$ is the vector of translational-respiratory-motion-corrected k-space samples, $U\in R^{N_{c}\left(\sum_{i=1}^{N_{b}}K_{i}\right)\times N_{c}N_{b}N}$ contains soft-gating respiratory bin weights between 0 and 1, $F\in C^{N_{c}N_{b}N\times N_{c}N_{b}N}$ applies the 3D Fourier transform for each coil and respiratory bin, $S\in C^{N_{c}N_{b}N\times N_{b}N}$ contains the 3D coil sensitivity maps, and * denotes the conjugate transpose. We note that except for the omission of $M\in R^{N_{b}N\times N}$, which acts to apply NR respiratory motion correction, this zero-filled reconstruction is equivalent to multiplying by the conjugate transpose of *E*, the encoding operator, which is defined as

(3) E=UFSM

and is later utilized in the DC step of the alternating MoCo-MoDL reconstruction.

#### 2.2.2 Deep-learning-based non-rigid motion estimation

Respiratory-bin magnitude 3D image patches, with the same dimensions as the 3D patch being reconstructed, were input pairwise to the DiRespME-Net [34] convolutional neural network, as depicted in Additional File 1(a). Each pair comprised the reference end-expiration respiratory bin and one of the three remaining bins. Since the input zero-filled bin images were strongly degraded by undersampling artifacts, an initial iterative SENSE [37] reconstruction with 10 iterations (selected following a limited optimization) was applied to each bin to create specific bin images solely for motion estimation. To reduce the computational load, these images were downsampled by a factor of two in each dimension following the SENSE reconstruction. The output of the network, a 3D velocity vector field representing the NR motion between the two input translational-motion-corrected respiratory bins, was integrated via a scaling and squaring layer, in the manner of Dalca et al. [33], [34], to produce a diffeomorphic motion field. Finally, each motion field was upsampled by a factor of two in each dimension to match the dimensions of the 3D image patches.

#### 2.2.3 Deep-learning-based iterative motion-corrected reconstruction

The final image was reconstructed using the motion-informed MoDL [35], [29] method, which alternates between a DC step and a regularization step, the latter being achieved via the application of the denoising U-Net depicted in Additional File 1(b). The iterative scheme sought to minimize

(4) $$L\left(\rho \right)={∥E\rho -b∥}_{2}^{2}+\lambda {∥\rho -D_{\theta }\left(\rho \right)∥}_{1}^{2}.$$

Here, $D_{\theta }$ represents the non-linear action of the denoising U-Net with parameters *θ* (learnt during training) and *λ* is a penalty weighting that determines the relative importance of the first term, which enforces DC, and the second term, which enforces the regularization of the reconstructed 3D image ***ρ***.

In the DC step, the first term in Eq. (4) was minimized by solving

(5) $$E^{*}E\rho =E^{*}b$$

with conjugate gradient descent. NR motion correction was explicitly incorporated via the encoding operator *E*, as defined in Eq. (3). Since the motion fields estimated by DiRespME-Net were diffeomorphic, both the forwards and inverse fields were available, facilitating the multiplications by *M* and M* which arise in the implementation of the conjugate gradient descent algorithm. Additionally, the inverse motion fields were applied to the input zero-filled image to yield

(6) $$M^{*}\rho_{ZF}=E^{*}b,$$

the required right-hand side of Eq. (5).

The real and complex components of the DC-step output image were then passed to the denoising U-Net, as depicted in Additional File 1(b). This step took the place of the low-rank patch-based denoising in an NR-PROST reconstruction [15] and acted to reduce noise and artifacts in the image. The output of the U-Net was taken as the starting guess in the next DC iteration, and the alternating sequence was repeated until a pre-determined number of iterations was reached.

### 2.3 Generation of training data

Fully sampled data sets were not acquired for the CHD patient cohort. Instead, each patient was scanned with the proposed sequence twice; once with three-or-four-fold undersampling and again with seven-fold undersampling. As such, an NR-PROST reconstruction framework was adopted to generate 3D reference images from the three-or-four-fold undersampled VD-CASPR acquisitions which could be treated as “ground-truth” images for network training.

**Fig. 3:**
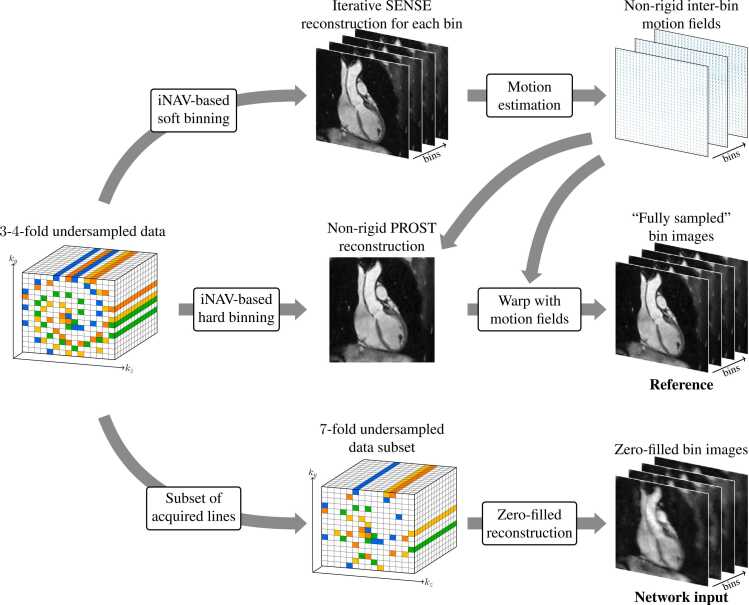
Schematic of the procedure used to generate training data from a three-or-four-fold undersampled acquisition. A set of reference 3D bin images and corresponding zero-filled 3D bin images with seven-fold undersampling are generated for each dataset. iNAV: image-based navigators, PROST: patch-based low-rank reconstruction.

The procedure for generating training data is depicted in Fig. 3. Initially, iNAV-based respiratory binning was applied to sort the acquired k-space data into four respiratory bins using soft-gating [36], [14] weights. An iterative sensitivity encoding (SENSE) [37] reconstruction was performed on each respiratory bin to generate bin images that could be used to estimate NR inter-bin respiratory motion fields using the software NiftyReg (Centre for Medical Image Computing, University College London, London, UK) [38]. These fields were then employed in an NR-PROST [15] reconstruction of the end-expiration-respiratory-bin image. To obtain a reference image for every respiratory bin, the NiftyReg motion fields were used to warp the end-expiration-bin NR-PROST image to each of the remaining bins, thus creating a set of reference bin images that were treated as fully sampled for training purposes.

**Fig. 4:**
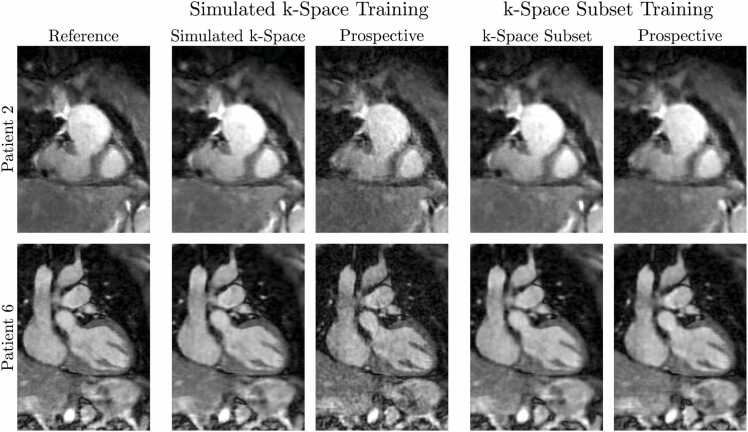
Comparison between the MoCo-MoDL reconstructions resulting from two different training-data-generation schemes, alongside reference images, for two example patients. For each scheme, the framework is applied to retrospectively undersampled data matching the data used in training (simulated-k-space data or k-space-subset data), and for both schemes the framework is also applied to prospectively undersampled data. Grain-like artifacts are evident when the simulated-data-trained network is applied to prospectively undersampled data. This is not the case using the proposed k-space-subset undersampling scheme. MoCo-MoDL, motion-corrected model-based deep learning

For the original training of the MoCo-MoDL framework for CMRA applications [29], fully sampled and two-to-three-fold undersampled data were used to reconstruct reference images, and the corresponding k-space data were generated synthetically from these images. In this study, training the network with data generated in such a manner was found to result in grain-like image artifacts in the final network reconstructions, as depicted in Fig. 4 for two example patients (not included in the training data). These artifacts were present when the network was used to reconstruct images from prospectively undersampled data, but not when it was used with synthetic k-space data generated in the same manner as the training data, suggesting some aspect of the data acquisition is not well-modeled by the synthetic data. To avoid this, the seven-fold undersampled k-space dataset corresponding to each set of fully sampled bin images was instead formed as a subset of the k-space readouts in the three-or-four-fold undersampled dataset, leading to the removal of the artifacts from prospective reconstructions, as seen in Fig. 4.

Simulating a seven-fold-undersampled VD-CASPR trajectory for the same heart rate would not guarantee that each of the acquired readouts was also acquired in the three-or-four-fold undersampled trajectory and taking a subset of the acquired VD-CASPR spiral interleaves would result in the center of the *k*_*y*_-*k*_*z*_ plane not being fully acquired. For this reason, a retrospective undersampling approach was taken that preserved the sampling density profile of a VD-CASPR trajectory despite losing the spiral-like profile order of the readouts in each heartbeat. First, the *k*_*y*_-*k*_*z*_ plane of each of the seven-fold undersampled prospective acquisitions was segmented into 20 elliptic annuli, and the percentage of the readouts located in each annulus that were acquired was calculated. Then, to implement the retrospective undersampling scheme, the *k*_*y*_-*k*_*z*_ planes of the three-or-four-fold undersampled k-space datasets were similarly segmented, and, within each annulus, a subset of acquired readouts was randomly selected, with the total number in each annulus chosen such that the acquisition percentage within that annulus matched the average of the prospectively undersampled datasets.

Eq. (2) was then applied to the retrospectively undersampled data to generate zero-filled bin images for network input, in the same manner as it was applied to prospectively undersampled data before input to the network.

### 2.4 Data acquisition

Forty-seven adult patients with CHD (20 female; age: 33 ± 13 years, range: 18–76 years) were recruited for the prospective study. The study was approved by the National Research Ethics Service (15/NS/0030) and written informed consent was obtained from each participant according to institutional guidelines.

Subjects were scanned on a 1.5T magnetic resonance imaging (MRI) scanner (MAGNETOM Aera, Siemens Healthcare, Erlangen, Germany) with the following parameters: FOV (with two-fold readout oversampling) = 400 mm × 300 mm × 72–108 mm, spatial resolution = 1.5 mm × 1.5 mm × 1.5 mm, flip angle = 90^∘^, TE = 1.75 ms, *T*_2_-preparation duration = 40 ms, coronal orientation.

Each subject was scanned twice using the bSSFP sequence described in Section 2.1; once with three-or-four-fold undersampling (6 with three-fold, 41 with four-fold, the latter being used consistently once it was established to provide good image quality) and again with seven-fold undersampling. We note that the reported undersampling factors are relative to a fully acquired G-CASPR [19] acquisition, which includes an elliptical shutter in k-space. Relative to the fully sampled rectangular *k*_*y*_-*k*_*z*_ plane, the undersampling factors would increase by a factor of 4/*π* from 3, 4, and 7 to 3.8, 5.1, and 8.9, respectively.

### 2.5 End-to-end training

The CHD patients were randomly sorted into a training set (39 patients) and a test set (8 patients). Of the training set, 5 patients were acquired with three-fold undersampling and 34 with four-fold undersampling. The networks were trained in an end-to-end manner on a 16 GB NVIDIA Quadro RTX 5000 graphics processing unit (GPU; NVIDIA Corporation, Santa Clara, California,) using the 39 datasets allocated to the training set. Each epoch, one training step was implemented for each subject in the training set, with a different 48 × 272 × 128-voxel patch randomly selected per epoch from the 272 × 272 × 128-voxel reference and zero-filled images. This patch size was selected to avoid GPU memory limitations during training.

The total training loss *L* was calculated as

(7) $$L=\lambda_{1}L_{mot}+\lambda_{2}L_{recon}+\lambda_{3}L_{reg},$$

where the motion loss *L*_*mot*_ was calculated as the Charbonnier loss between the end-expiration-bin reference image and the warped reference images, plus a smoothness loss on the motion fields, the reconstruction loss *L*_*recon*_ was calculated as the Charbonnier loss between the reference image and the MoCo-MoDL alternating reconstruction and the regularization loss *L*_*reg*_ was calculated as the *l*^2^-norm of the convolution kernel parameters in both networks. The penalty weights multiplying the losses in Eq. (7) were set to *λ*_1_ = 100, *λ*_2_ = 10, and *λ*_3_ = 1 following a limited hyper-parameter search based on visual inspection of the output images. Other parameters for the end-to-end training, set following the hyper-parameter search, were: no. of epochs = 1600 (leading to a training time of ∼103 hours), initial learning rate = 3 × 10^−4^, learning rate decreased by a factor of two every 400 epochs, no. of MoDL iterations = 5 (4 U-Net passes), and no. of conjugate gradient iterations per DC step = 3.

### 2.6 Reconstruction

After training, the MoCo-MoDL reconstruction framework was applied to the seven-fold prospectively undersampled datasets of the eight patients allocated to the test set. The framework was also applied to retrospectively undersampled datasets of the same eight patients, formed from the three-or-four-fold undersampled acquisitions of those patients using the same method previously applied to generate training data. Of these acquisitions, one was acquired with three-fold undersampling and seven were acquired with four-fold undersampling.

The framework was applied on a patch-wise basis to overlapping 48 × 272 × 128-voxel patches, matching the patch size used in training. To avoid edge effects, a five-voxel layer was removed from the edges of each patch in the *x* (patch) direction, leaving a remaining overlap of four voxels.

For comparison, the NR-PROST [15] method, as applied to the three-or-four-fold undersampled acquisitions to generate reference images, was also applied to both the prospectively and retrospectively undersampled datasets for each of the patients in the test set.

### 2.7 Analysis

In the case of retrospective undersampling, an inherently co-registered 3D reference image was available for the calculation of quantitative reconstruction quality metrics. Thus, for the eight patients in the test set, the mean squared error (MSE) and structural similarity index measure (SSIM) were calculated for both the MoCo-MoDL and NR-PROST reconstructions in a manually selected cuboid region of interest around the heart.

Statistical significance for these metrics was evaluated using a paired samples t-test between the NR-PROST and MoCo-MoDL reconstructions of the retrospectively undersampled data.

For reconstructions of the prospectively undersampled data, no inherently co-registered reference images were available and thus quantitative error metrics were not applicable. Visual image quality was assessed by a cardiologist (A.F., 5 years of experience in cardiac MRI, European Association for Cardiovascular Imaging (EACVI) accreditation level III) for the eight test-set patients on each of the non-co-registered reference, NR-PROST and MoCo-MoDL whole-heart images. For this assessment, the reviewer was blinded to the reconstruction method and participant characteristics. The reviewer independently scored the image quality of all intrapericardial structures using a five-point Likert scale. For each structure, one score was allocated for sharpness of vessel or cardiac wall borders (1: non-diagnostic; 5: excellent) and another for extent of artifacts (1: severe artifacts; 5: minimal artifacts). Additionally, the reviewer scored their diagnostic confidence to perform sequential segmental analysis with each dataset using a four-point Likert scale (1: low confidence, 2: moderate, but additional imaging required, 3: high (diagnostic), 4: definite). After grading the diagnostic confidence, the CMR findings could be adjudicated with locally available echocardiographic, catheterization, computed tomography (CT), and operative data. Image analysis and reformatting were performed with the freely available software Horos (version 1.1.7).

Statistical significance for the visual image quality scores was evaluated using a Wilcoxon signed rank test between the reference images and the NR-PROST and MoCo-MoDL reconstructions of the prospectively undersampled data. A *p* value of less than 0.05 was regarded as statistically significant.

## 3 Results

The MoCo-MoDL reconstruction framework was successfully applied to each of the seven-fold prospectively undersampled data sets. Acquisition times were 2.1 ± 0.3 minutes, while reconstruction times were ∼30 seconds, a speed-up of approximately 240-fold relative to NR-PROST.

### 3.1 Retrospective undersampling

**Fig. 5:**
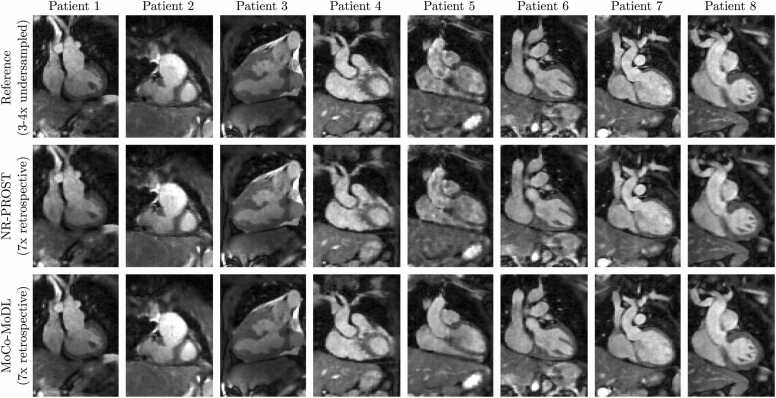
Coronal 2D slices of 3D whole-heart reference images (top row), NR-PROST reconstructions of retrospectively undersampled data (middle row), and MoCo-MoDL reconstructions of retrospectively undersampled data (bottom row) for the eight patients in the test set. Each image is individually normalized. MoCo-MoDL, motion-corrected model-based deep-learning; NR-PROST, non-rigid motion-corrected patch-based low-rank reconstruction method.

Fig. 5 depicts 2D coronal slices of the 3D whole-heart reconstructions of each of the retrospectively undersampled test-patient datasets, using NR-PROST (middle row) and the proposed MoCo-MoDL framework (bottom row), alongside the reference images reconstructed from three-or-four-fold undersampled datasets (top row). Similar image quality to that of the reference images is seen in both the NR-PROST and MoCo-MoDL reconstruction for most subjects. By visual inspection, the most apparent difference is seen for patient 5, where both the reference image and NR-PROST reconstruction appear to have “blotch-like” artifacts across the entire image which are not evident in the MoCo-MoDL reconstruction.

Difference maps for the reconstructions of retrospectively undersampled data are presented in Additional File 2. These depict the differences between the (normalized) reference-image slices in Fig. 5 and the images produced by the two reconstruction methods (with the reference-image-normalization scaling factors applied). They demonstrate that the differences between the reference images and the proposed MoCo-MoDL framework are of a similar magnitude to those obtained when the NR-PROST technique is applied to the retrospectively undersampled data. Additionally, the differences appear to be largely noise-like, rather than structural, in nature.

**Fig. 6:**
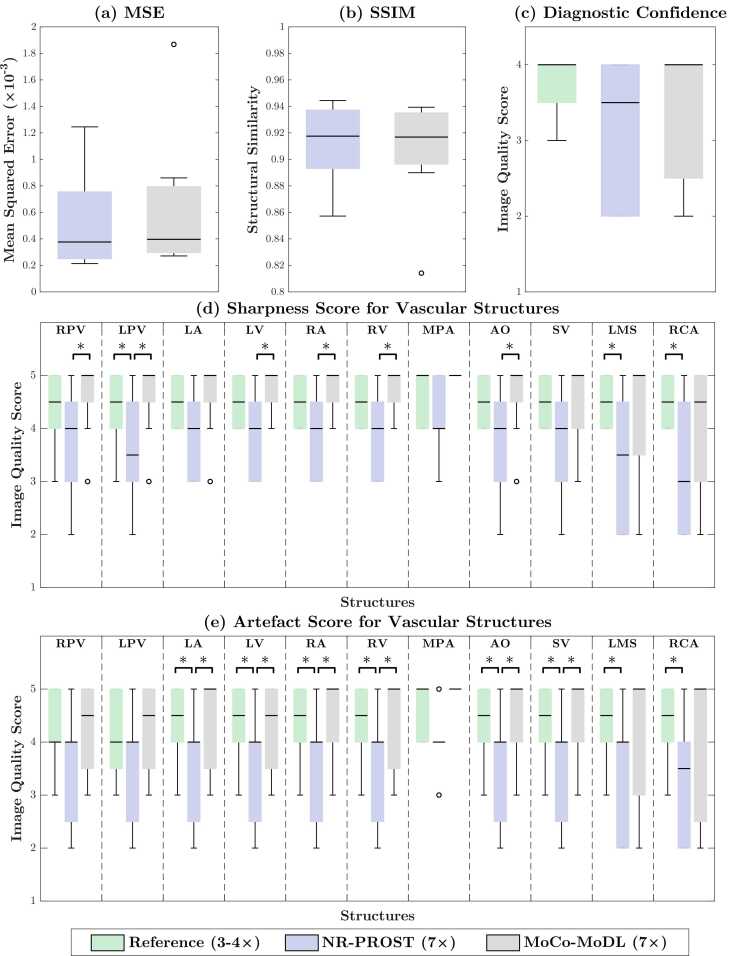
Box plots of quantitative (a), (b) and qualitative (c)-(e) metrics of image quality measured for the eight test-set patients. Quantitative metrics MSE (a) and SSIM (b) are calculated relative to the 3D reference images. Qualitative measures of diagnostic confidence (c) are assessed on a scale from 1 to 4, while sharpness (d) and artifact level (e) are assessed on a scale from 1 to 5. Statistical significance (*p* < 0.05) is denoted by * in each plot. The sharpness and artifact scores in the main pulmonary artery (MPA) are only calculated for six patients, since the two other patients have no MPA in view of their underlying diagnoses. AO, aorta; LA, left atrium; LMS, left main stem; LPV, left pulmonary vein; LV, left ventricle; MoCo-MoDL, motion-corrected model-based deep-learning; MPA, main pulmonary artery; MSE, mean squared error; NR-PROST, non-rigid motion-corrected patch-based low-rank reconstruction method; RA, right atrium; RCA, right coronary artery; RPV, right pulmonary vein; RV, right ventricle; SSIM, structural similarity index measure; SV, superior vena cava.

Quantitative error metrics for the retrospectively undersampled reconstructions, relative to the reference images, are presented in Fig. 6a and b. The mean MSE for NR-PROST of 5.28 × 10^−4^ is lower than the mean MoCo-MoDL value of 6.39 × 10^−4^. The mean SSIM values for the two methods are comparable, with the NR-PROST value of 0.912 slightly higher than the MoCo-MoDL value of 0.906. However, no statistically significant difference in either MSE or SSIM is observed (*p* = 0.18 and *p* = 0.32, respectively). We note that patient 5, previously discussed as exhibiting the clearest differences between NR-PROST and MoCo-MoDL, records both the lowest MSE and the highest SSIM values for both reconstruction methods, and is the outlier seen in both MoCo-MoDL box plots.

### 3.2 Prospective undersampling

**Fig. 7:**
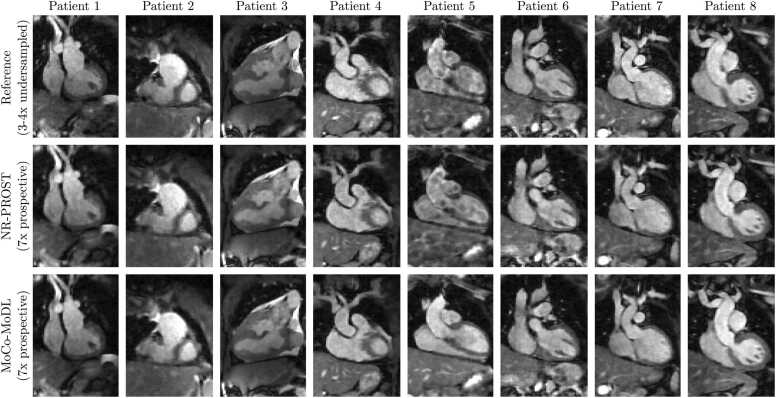
Coronal 2D slices of 3D whole-heart reference images (top row), NR-PROST reconstructions of prospectively undersampled data (middle row), and MoCo-MoDL reconstructions of prospectively undersampled data (bottom row) for the eight patients in the test set. Note that the reference image is obtained from the three-or-four-fold undersampled scans which preceded the seven-fold undersampled prospective scans, and so is not inherently co-registered with the prospective reconstructions. Each image is individually normalized. MoCo-MoDL, motion-corrected model-based deep learning; NR-PROST, non-rigid motion-corrected patch-based low-rank reconstruction method.

**Fig. 8:**
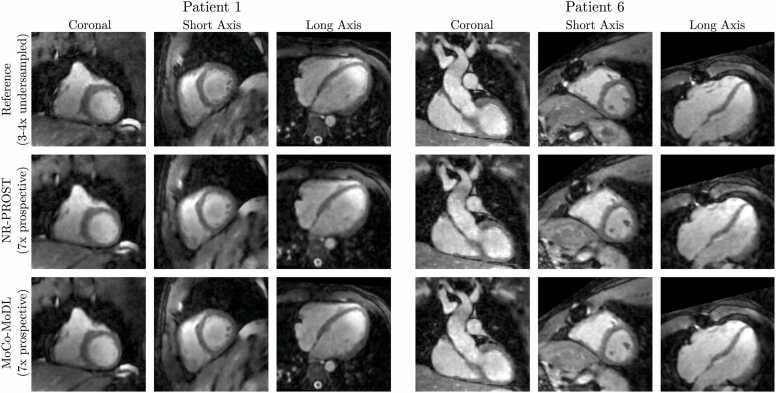
Coronal and oblique short- and long-axis slices of 3D whole-heart reference images (top row), NR-PROST reconstructions of prospectively undersampled data (middle row), and MoCo-MoDL reconstructions of prospectively undersampled data (bottom row) for two example patients from the test set. The oblique slices are obtained via interpolation of the isotropic-resolution 3D image. Note that the reference image is obtained from the three-or-four-fold undersampled scans which preceded the seven-fold undersampled prospective scans, and so is not inherently co-registered with the prospective reconstructions. Each image is individually normalized. MoCo-MoDL, motion-corrected model-based deep learning; NR-PROST, non-rigid motion-corrected patch-based low-rank reconstruction method.

Coronal slices of the 3D whole-heart images reconstructed from prospectively undersampled data are presented in Fig. 7 for each patient in the test set. The MoCo-MoDL reconstruction quality appears visually to be similar to that seen for the retrospectively undersampled data. Additionally, oblique slices in short-axis and long-axis orientations are shown for two example patients in Fig. 8. These demonstrate the benefits of acquiring 3D whole-heart images with isotropic spatial resolution; 2D slices with arbitrary orientations can be generated from the 3D images via interpolation.

Qualitative image quality scores for the reference images and two reconstruction methods are presented in Fig. 6c-e. The overall diagnostic confidence for each image, on a scale of 1 to 4, is plotted in Fig. 6c. No statistically significant difference is observed between the reference (three-or-four-fold undersampled) images and either of the reconstruction methods applied to the seven-fold prospectively undersampled data. We note that in the case that exhibited the clearest differences between NR-PROST and MoCo-MoDL, patient 5, a diagnostic confidence score of 2 was awarded for the artifact-affected NR-PROST reconstruction, indicating additional imaging would be required, while the MoCo-MoDL image achieved a score of 4, indicating a definite diagnosis would be possible.

The image sharpness scores, assessed on 11 vascular structures on a scale of 1 to 5, are plotted in Fig. 6d. For 10 of the 11 structures, MoCo-MoDL recorded a median score of 5; a median score of 4.5 was recorded on the remaining structure, the right coronary artery. In 9 structures, this was the highest median score of the three images, while in the remaining two it was the equal highest median alongside the reference image. For all structures, the NR-PROST reconstructions recorded the lowest sharpness scores. The difference between the reference-image and NR-PROST sharpness scores was statistically significant for three structures, while the difference between the MoCo-MoDL and NR-PROST scores was statistically significant for six structures. No statistically significant differences were seen between the sharpness of the reference and MoCo-MoDL images for any structures.

The artifact scores, assessed for the same 11 vascular structures on a scale of 1 to 5, are presented in Fig. 6e. Again, the NR-PROST prospective reconstructions record the lowest (or equal-lowest) median score across every structure. For nine structures, MoCo-MoDL recorded the highest median score, and for the two remaining structures, the reference and MoCo-MoDL median scores were equal. The difference in artifact scores between reference and NR-PROST was statistically significant for eight structures, and the difference between MoCo-MoDL and NR-PROST was statistically significant for six structures.

### 3.3 Clinical applicability

**Fig. 9:**
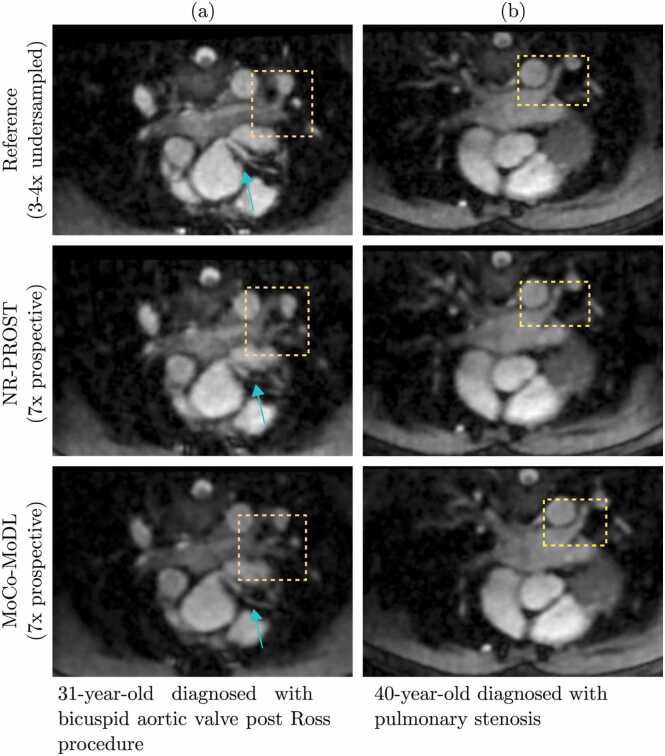
Multiplanar reformatted images from two adult congenital heart disease patients. (a) A 31-year-old male was diagnosed with bicuspid aortic valve post-Ross procedure. The connections to the right-sided pulmonary veins and the course of the main stem are well-demarcated with the proposed approach. (b) A 40-year-old female was diagnosed with pulmonary stenosis, illustrating comparable image quality in the depiction of the pulmonary veins to left atrium. For both patients, the NR-PROST and MoCo-MoDL reconstructions were applied to prospectively undersampled data. MoCo-MoDL, motion-corrected model-based deep-learning; NR-PROST, non-rigid motion-corrected patch-based low-rank reconstruction method.

**Fig. 10:**
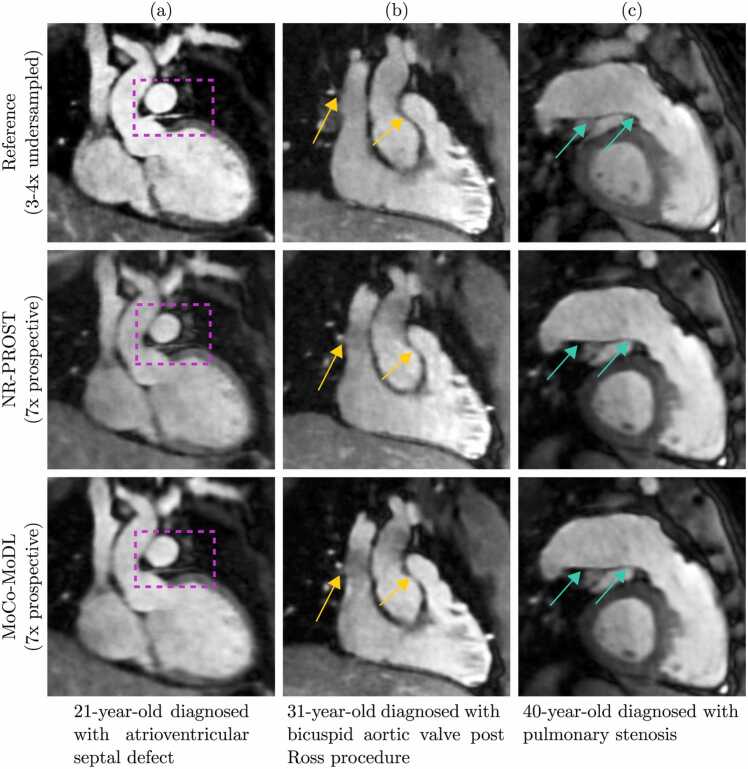
Multiplanar reformatted images from three adult congenital heart disease patients. (a) A 21-year-old female was diagnosed with atrioventricular septal defect. The connection and proximal course of the left anterior descending coronary artery are well-demarcated (purple boxes). (b) A 31-year-old male was diagnosed with bicuspid aortic valve post-Ross procedure, demonstrating the connections to the pulmonary artery and systemic vein (yellow arrows). (c) A 40-year-old female diagnosed with pulmonary stenosis demonstrated the connections from the right ventricle to the main and dilated right pulmonary artery (turquoise arrows). For all patients, the NR-PROST and MoCo-MoDL reconstructions were applied to prospectively undersampled data. MoCo-MoDL, motion-corrected model-based deep-learning; NR-PROST: non-rigid motion-corrected patch-based low-rank reconstruction method.

The cardiac connections and thoracic vasculature were clearly delineated with MoCo-MoDL, as demonstrated in the multiplanar reformatted images seen in Figs. 9 and 10. A comparison of the MoCo-MoDL to the reference images and NR-PROST reconstructions illustrates that MoCo-MoDL recovers smaller vascular structures, such as the pulmonary veins (Fig. 9a and b) and coronary artery (Fig. 9a), along with sequential cardiovascular segments with good image quality.

## 4 Discussion

We have adapted, trained, and validated a MoCo-MoDL reconstruction framework for an adult CHD patient cohort. 3D whole-heart images were achieved from ∼2-minute scans with a ∼30-second reconstruction, the latter representing a ∼240-fold speed-up relative to NR-PROST reconstruction times.

The framework was trained on a 39-patient training set against reference data generated from three-or-four-fold undersampled acquisitions and tested on the 8-patient test set on seven-fold undersampled acquisitions. To avoid the artifacts that can result from a mismatch between the training data and the prospectively undersampled data to which the framework was applied (Fig. 4), a k-space undersampling scheme with realistic VD-CASPR sampling-density profiles was employed. 3D whole-heart images with comparable quality to reference images were reconstructed using both retrospectively undersampled (Fig. 5) and prospectively undersampled (Fig. 7) data.

Expert image quality scores, assessed for overall diagnostic confidence and for image sharpness and artifact level across 11 vascular structures (Fig. 6), consistently showed that the MoCo-MoDL reconstruction outperformed the NR-PROST technique. The sharpness and artifact scores of MoCo-MoDL were higher than those of NR-PROST, with statistical significance, for 12 of the 22 metrics. The differences seen in the 10 other metrics were not found to be statistically significant. The image quality achieved by MoCo-MoDL was found to be generally comparable to that of the reference images, with no statistically significant differences observed. Both achieved a median diagnostic confidence score of 4 (on a 4-point scale), MoCo-MoDL recorded the highest median score for 18 of the 22 structure-specific metrics and equal medians were obtained for the remaining 4 metrics.

The quantitative error metrics MSE and SSIM revealed no statistically significant difference between the proposed MoCo-MoDL approach and the state-of-the-art NR-PROST method. However, it should be noted that these metrics were calculated with comparison to the reference images, and the reference images were reconstructed using NR-PROST from three-or-four-fold undersampled data. Thus, they do not represent an ideal fully sampled image and could be expected to share certain image qualities with the prospective NR-PROST reconstruction.

The reported MoCo-MoDL reconstruction speed-up of ∼240-fold was achieved using the same 16 GB GPU as used for training, while the NR-PROST reconstructions were performed offline on a CPU. Data pre-processing and network loading times were not included in the MoCo-MoDL inference time.

## 5 Limitations

Limitations of this study include the relatively small size of the test set. Deep-learning-based methods tend to improve when they are trained on larger training datasets and thus a trade-off between the size of the training and test sets exists when selecting the number of patients to be randomly allocated to each set; increasing the efficacy of the network may come at the cost of reducing the statistical power of the analyses.

A further limitation is that all acquired training and test data were acquired using the same MRI scanner; the generalizability of the network to different scanners, field strengths, and spatial resolutions was not assessed and should be investigated in future work.

## 6 Conclusions

In this study, a deep-learning-based motion-corrected reconstruction framework, MoCo-MoDL, was successfully applied to a cohort of adult CHD patients to reconstruct 3D whole-heart images from seven-fold undersampled acquisitions. Image quality comparable to that of the state-of-the-art motion-corrected NR-PROST reconstruction of three-to-four-fold undersampled data was achieved with scan times of 2.1 ± 0.3 minutes and reconstruction times of ∼30 seconds.

## Funding

The authors acknowledge financial support from (1) King’s BHF Centre for Award Excellence PG/18/59/33955 and RG/20/1/34802, (2) EPSRC EP/V044087/1, EP/P001009/1, EP/P032311/1, EP/P007619, (3) Wellcome EPSRC Centre for Medical Engineering (NS/A000049/1), (4) Millennium Institute for Intelligent Healthcare Engineering ICN2021_004, FONDECYT 1210637 and 1210638, (5) IMPACT, Center of Interventional Medicine for Precision and Advanced Cellular Therapy, Santiago, Chile. ANID-Basal funding for Scientific and Technological Center of Excellence, IMPACT, #FB210024 (6) the Department of Health through the National Institute for Health Research (NIHR) comprehensive Biomedical Research Centre award, (7) NIHR Cardiovascular MedTech Co-operative, (8) the Technical University of Munich - Institute for Advanced Study and (9) the Government of Denmark. The views expressed are those of the authors and not necessarily those of the BHF, NHS, NIHR, or the Department of Health.

## Author contributions

**Claudia Prieto:** Writing – review and editing, Supervision, Funding acquisition, Conceptualization. **René M. Botnar:** Writing – review and editing, Supervision, Funding acquisition, Conceptualization. **Haikun Qi:** Writing – review and editing, Software. **Thomas J. Fletcher:** Writing – review and editing, Software, Methodology. **Lina Felsner:** Writing – review and editing, Software, Methodology. **Anastasia Fotaki:** Writing – review and editing, Writing – original draft, Visualization, Investigation, Formal analysis, Data curation. **Andrew Phair:** Writing – review and editing, Writing – original draft, Visualization, Software, Methodology, Investigation, Formal analysis, Conceptualization.

## Ethics approval and consent

The study was approved by the National Research Ethics Service (15/NS/0030) and written informed consent was obtained from each participant according to institutional guidelines.

## Consent for publication

All participants gave written informed consent for publication.

## Declaration of competing interests

The authors declare that they have no known competing financial interests or personal relationships that could have appeared to influence the work reported in this paper.

## Data Availability

The datasets generated and analyzed in this study are available from the authors upon reasonable request.
