# Supplementary material for: A motion-corrected deep-learning reconstruction framework for accelerating whole-heart magnetic resonance imaging in patients with congenital heart disease
Source: J Cardiovasc Magn Reson. 2024 Mar 22;26(1):101039. doi: 10.1016/j.jocmr.2024.101039 (PMC10993190; doi:10.1016/j.jocmr.2024.101039)
Supplement: Supplementary file 1 — Supplementary material [file mmc1.pdf]

8 Additional Files

8.1 Additional File 1

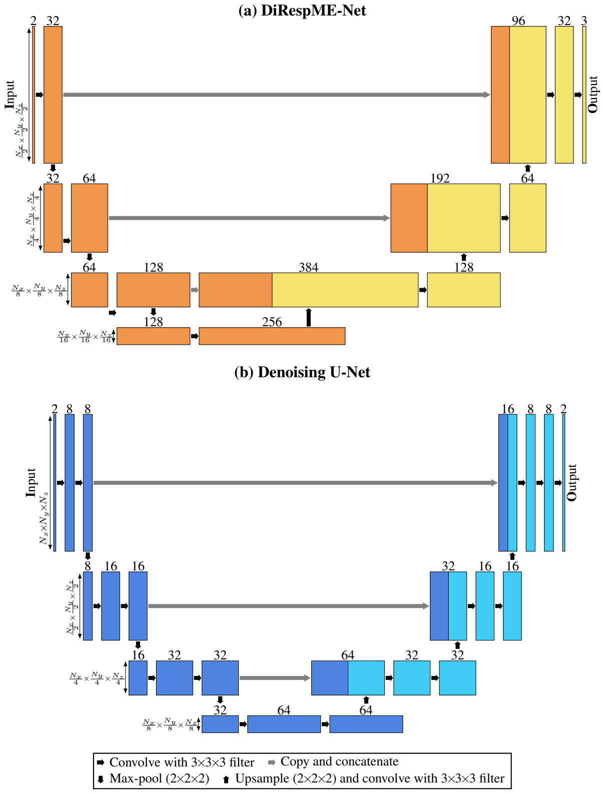

File name:
Title: Network architecture diagrams
Description of data: Architecture of (a) the diffeomorphic motion estimation network (DiRespME-Net) and (b) the denoising U-Net. In (a), two absolute-value image patches, with size $\frac{N_x}{2} \times \frac{N_y}{2} \times \frac{N_z}{2}$, from different motion bins are inputted to the network. The output is a velocity vector field of the same size, which passes through a scaling and squaring integration layer to produce the non-rigid motion field. In (b), the

real and complex components of an image patch (of size $N_x \times N_y \times N_z$) are inputted as separate layers, and the output image patch of the same size similarly contains both real and complex layers. For both networks, every convolution is followed by non-linear ReLU (rectified linear unit) activation, with the exception of the final convolution before output, which has linear activation. For the denoising U-Net only, bias is included in the activation function.

8.2 Additional File 2

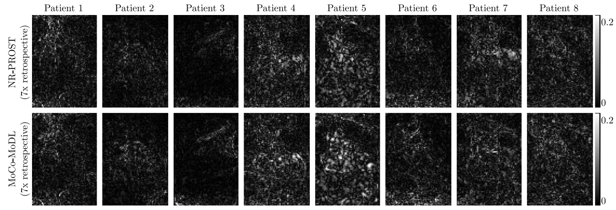

File name:
Title: Difference maps
Description of data: Coronal 2D slices of 3D difference maps between reference images and reconstructions of retrospectively undersampled data performed using NR-PROST (top row) and MoCo-MoDL (bottom row), for each of the eight patients in the test set. In each case, a scaling factor which normalises the reference-image slice is applied to both images before the difference map is calculated. The slices presented match those of the reconstructions displayed in Figure 5.
